# Sequence variation data of the mitochondrial DNA D-loop region of the captive Malayan Gaur (*Bos gaurus hubbacki*)

**DOI:** 10.1016/j.dib.2018.11.117

**Published:** 2018-11-27

**Authors:** Badrul Munir Md-Zain, Aqilah Abdul-Aziz, Nor Rahman Aifat, Nur Syafika Mohd-Yusof, Nadiatur Akmar Zulkifli, Jeffrine Rovie Ryan Japning, Norsyamimi Rosli, Salmah Yaakop

**Affiliations:** a School of Environmental and Natural Resource Sciences, Faculty of Science and Technology, Universiti Kebangsaan Malaysia, 43600 Bangi, Selangor, Malaysia; b Department of Wildlife and National Parks (DWNP), KM10 Jalan Cheras, 56100 Kuala Lumpur, Malaysia

**Keywords:** Malayan gaur, *Bos gaurus hubbacki*, Seladang, Genetic variation

## Abstract

This article contains data of the sequence variation in the mitochondrial DNA D-loop region of the Malayan gaur (*Bos gaurus hubbacki*), locally known as the seladang, from two captive centers. Thirty fecal samples of Malayan gaur were collected from Jenderak Selatan Wildlife Conservation Center (Pahang) and the Sungkai Wildlife Reserve (Perak) for DNA extraction and amplification with polymerase chain reactions. DNA sequences were then analyzed using neighbor joining (NJ) and maximum parsimony (MP) methods. Based on the 652 base pairs obtained, we found seven variable characters with a value of 1%. The genetic distance between the two captive centers was 0.001. Haplotype analyses detected only four haplotypes between these two captive centers. Both NJ and MP trees demonstrate that all individuals in the Jenderak and Sungkai captive centers are in the same clade. Genetic variation of the Malayan gaur in these centers is considered low, possibly because individuals share the same common parent. This sequence variation data are of paramount importance for designing a proper breeding and management program of the Malayan gaur in the future.

**Specifications table**

**Table:** 

Subject area	Molecular Systematics, Genetics and Conservation Science
More specific subject area	Molecular Phylogeny
Type of data	Tables, figures
How data was acquired	Fecal DNA sampling and PCR using Eppendorf thermal cycler
Data format	Analyzed
Experimental factors	Phylogenetic analysis, bootstrap test
Experimental features	Molecular data was analysed in BioEdit Sequence Alignment Editor 7.2.0, ClustalW2 and MEGA 4.0
Data source location	Jenderak Selatan Wildlife Conservation Center (Pahang), Sungkai Wildlife Reserve (Perak) in Malaysia
Data accessibility	With this article

**Value of the data**

• The data presented here provide sequence variations of Malayan gaur in captivity.
• Genetic relationships among captive Malayan gaur individuals.
• Revealing inbreeding in which they might share the same common parent.
• The data are useful for decision-making in the breeding and management program of the Malayan gaur.

## 1 Data

**Fig. 1:**
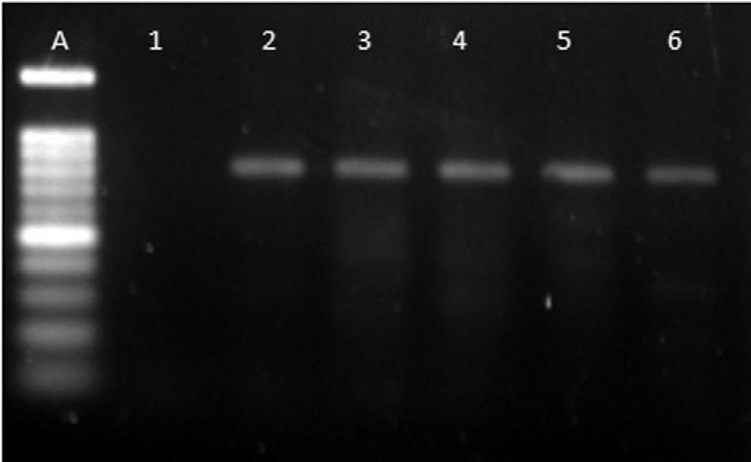
Result of the PCR process with an 800-base pair product. A = 100 base pairs; 1 = negative control; 2–6 = PCR products of the Malayan gaur.

We present D-loop region sequence data [1] for 33 individuals of Malayan gaur from Jenderak Selatan Wildlife Conservation Center, Pahang and from Sungkai Wildlife Reserve (Perak). We also provided forward and reverse primers [2] (Table 1) that were utilized in the polymerase chain reaction [3], initial concentration and volumes for each PCR reagent (Table 2) and PCR cycle profile for the D-loop region (Table 3). Amplification products were visualized as indicated in Fig. 1.

**Table 1:** Design of the primer pair for the D-loop region.

Primer	Sequence 5′-3′
Walid F	TCA CCG TCA ACT CCC AAA GCT GA
Walid R	AGG GGG AAG TTT TAT GGA AGG GGG

**Table 2:** Initial concentration and volumes for each PCR reagent.

PCR component	Final concentration	Volume (µl)
Distilled water (ddH_2_O)	–	18.8
10X PCR buffer	1X	2.5
dNTP mix (10 mM)	0.28 mM	0.7
MgCl2 (50 mM)	2.4 mM	1.2
Forward primer (10 µM)	0.12 uM	0.3
Reverse primer (10 µM)	0.12 uM	0.3
Taq Polymerase (5 U/µl)	1 U	0.2
DNA template	50 ng/uL	1.0
**Total**	–	25.0

**Table 3:** PCR cycle profile for the D-loop region.

PCR protocol	Temperature (°C)	Duration (s)	Cycle
Initial denaturation	94	180	–
Denaturation	94	60	35
Annealing	58	30	
Extension	72	90	
Post-extension	72	420	–
Incubation	4	∞	–

## 2 Experimental design, materials, and methods

**Fig. 2:**
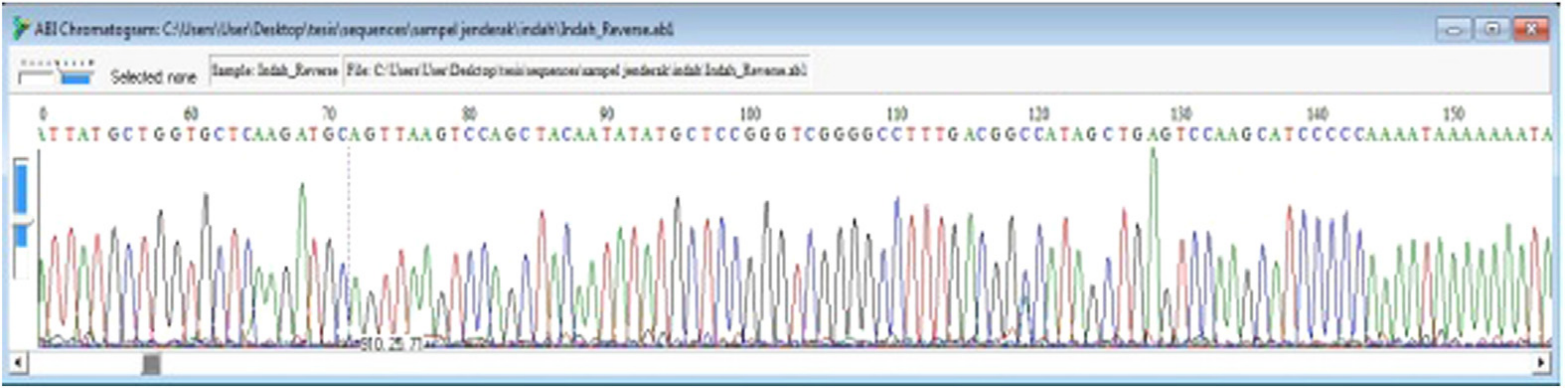
DNA chromatogram for PCR product of the D-loop region.

**Fig. 3:**
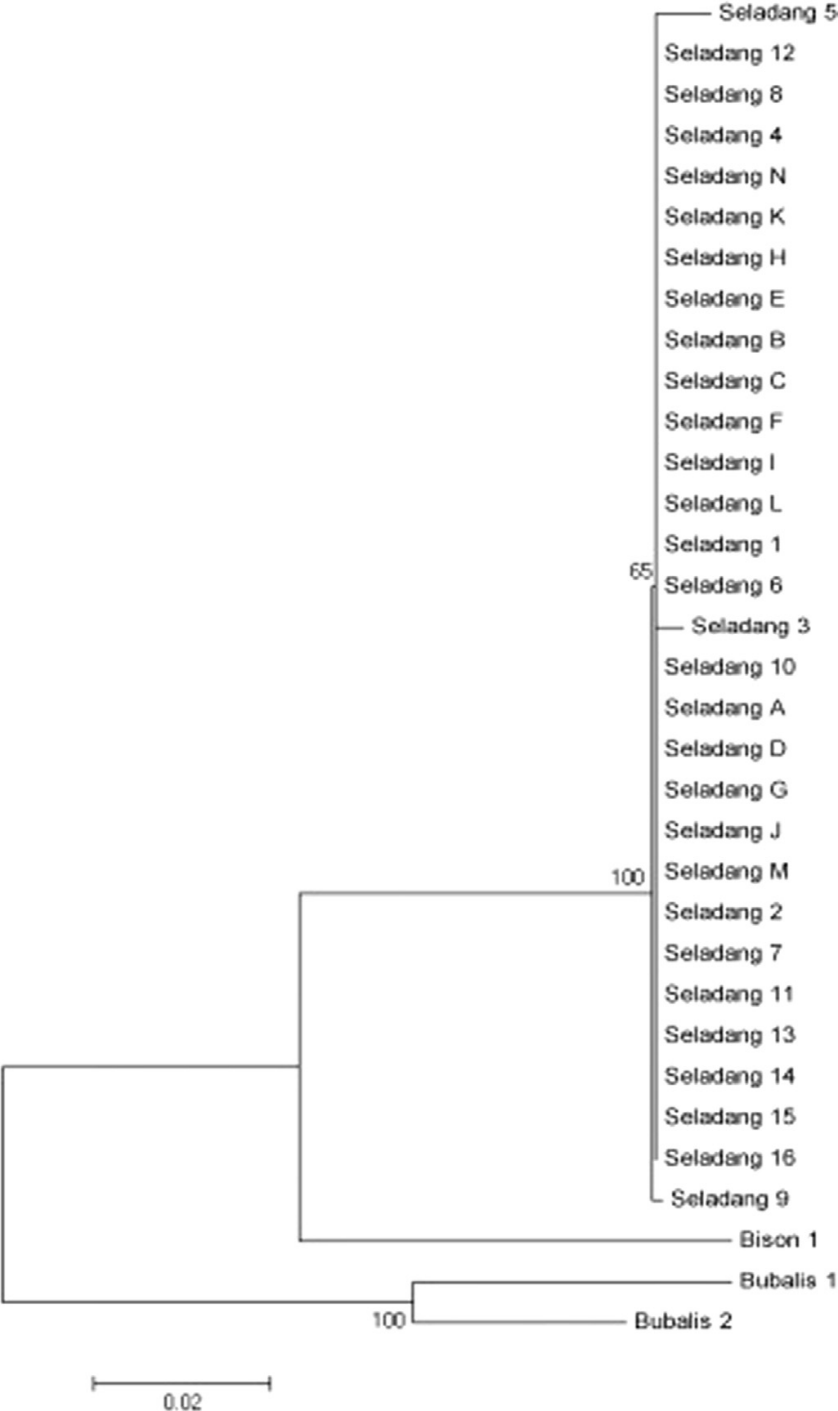
The neighbor joining phylogenetic tree estimated using the Kimura 2-parameter algorithm and 1000 bootstrap replications.

**Fig. 4:**
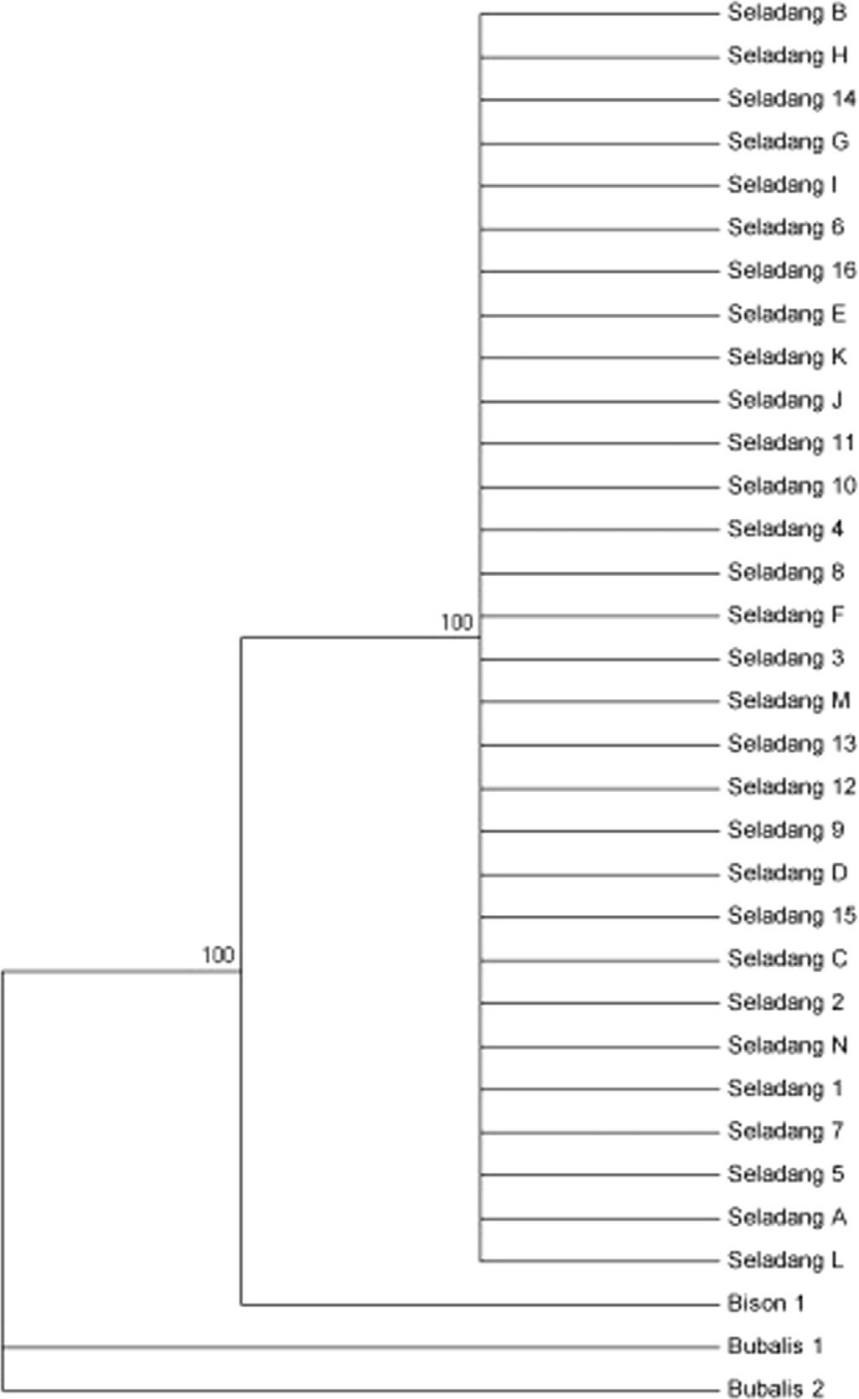
The maximum parsimony phylogenetic tree estimated using 1000 bootstrap replications.

The DNA chromatograms (Fig. 2) of the sequenced samples were visually checked using a BioEdit sequence alignment editor [4]. All DNA sequences were aligned (see Supplemental data) and edited using the ClustalW multiple alignment algorithm in Mega 4.0 software to achieve multiple sequence alignment [5]. Any sequence that varied by one or more nucleotides was considered a different haplotype (Table 4). All sequences were analyzed using PAUP 4.0b10 software for phylogenetic reconstruction [6]. We used two methods of analysis in PAUP: first, neighbor joining with the Kimura 2-parameter model [7] to reconstruct a neighbor joining phylogram (Fig. 3) and calculate the genetic distance (Table 5), second, a maximum parsimony analysis with stepwise additions (1000 replicates) in a heuristic search [8] and 50% majority rule consensus (Fig. 4). All trees were subjected to a bootstrap analysis with 1000 replicates to find bootstrap value support [9].

**Table 4:** Malayan gaur haplotype structure.

Haplotype	Haplotype sequence	Individual number	Captive site
Hap_1	CTCCCCC	27	14-Jenderak, 13-Sungkai
Hap_2	TTCCTCC	1	Sungkai (Seladang 3)
Hap_3	CTATCAT	1	Sungkai (Seladang 5)
Hap_4	CACCCCC	1	Sungkai (Seladang 9)

**Table 5:** Genetic distance value of the Malayan gaur between Sungkai and Jenderak.

Captive site	Jenderak	Sungkai
Jenderak	–	–
Sungkai	0.001	–
