# Supplementary material for: Sequence variation data of the mitochondrial DNA D-loop region of the captive Malayan Gaur (Bos gaurus hubbacki)
Source: Data Brief. 2018 Nov 27;24:103532. doi: 10.1016/j.dib.2018.11.117 (PMC6531834; doi:10.1016/j.dib.2018.11.117)
Supplement: Supplementary file 2 — Supplementary material. [file mmc2.doc]

**D-loop region sequence alignment**

....|....| ....|....| ....|....| ....|....| ....|....| ....|....|

5 15 25 35 45 55

Seladang_A CGCAAAGAGC CTTACCAGTA TTAAATTTAT CAAAAATTTA AATAACTCAA CACAGACTTT

Seladang_B CGCAAAGAGC CTTACCAGTA TTAAATTTAT CAAAAATTTA AATAACTCAA CACAGACTTT

Seladang_C CGCAAAGAGC CTTACCAGTA TTAAATTTAT CAAAAATTTA AATAACTCAA CACAGACTTT

Seladang_D CGCAAAGAGC CTTACCAGTA TTAAATTTAT CAAAAATTTA AATAACTCAA CACAGACTTT

Seladang_E CGCAAAGAGC CTTACCAGTA TTAAATTTAT CAAAAATTTA AATAACTCAA CACAGACTTT

Seladang_F CGCAAAGAGC CTTACCAGTA TTAAATTTAT CAAAAATTTA AATAACTCAA CACAGACTTT

Seladang_G CGCAAAGAGC CTTACCAGTA TTAAATTTAT CAAAAATTTA AATAACTCAA CACAGACTTT

Seladang_H CGCAAAGAGC CTTACCAGTA TTAAATTTAT CAAAAATTTA AATAACTCAA CACAGACTTT

Seladang_I CGCAAAGAGC CTTACCAGTA TTAAATTTAT CAAAAATTTA AATAACTCAA CACAGACTTT

Seladang_J CGCAAAGAGC CTTACCAGTA TTAAATTTAT CAAAAATTTA AATAACTCAA CACAGACTTT

Seladang_K CGCAAAGAGC CTTACCAGTA TTAAATTTAT CAAAAATTTA AATAACTCAA CACAGACTTT

Seladang_L CGCAAAGAGC CTTACCAGTA TTAAATTTAT CAAAAATTTA AATAACTCAA CACAGACTTT

Seladang_M CGCAAAGAGC CTTACCAGTA TTAAATTTAT CAAAAATTTA AATAACTCAA CACAGACTTT

Seladang_N CGCAAAGAGC CTTACCAGTA TTAAATTTAT CAAAAATTTA AATAACTCAA CACAGACTTT

Seladang_1 CGCAAAGAGC CTTACCAGTA TTAAATTTAT CAAAAATTTA AATAACTCAA CACAGACTTT

Seladang_2 CGCAAAGAGC CTTACCAGTA TTAAATTTAT CAAAAATTTA AATAACTCAA CACAGACTTT

Seladang_3 CGCAAAGAGC CTTACCAGTA TTAAATTTAT CAAAAATTTA AATAACTCAA CACAGACTTT

Seladang_4 CGCAAAGAGC CTTACCAGTA TTAAATTTAT CAAAAATTTA AATAACTCAA CACAGACTTT

Seladang_5 CGCAAAGAGC CTTACCAGTA TTAAATTTAT CAAAAATTTA AATAACTCAA CACAGACTTT

Seladang_6 CGCAAAGAGC CTTACCAGTA TTAAATTTAT CAAAAATTTA AATAACTCAA CACAGACTTT

Seladang_7 CGCAAAGAGC CTTACCAGTA TTAAATTTAT CAAAAATTTA AATAACTCAA CACAGACTTT

Seladang_8 CGCAAAGAGC CTTACCAGTA TTAAATTTAT CAAAAATTTA AATAACTCAA CACAGACTTT

Seladang_9 CGCAAAGAGC CTTACCAGTA TTAAATTTAT CAAAAATTTA AATAACTCAA CACAGACTTT

Seladang_10 CGCAAAGAGC CTTACCAGTA TTAAATTTAT CAAAAATTTA AATAACTCAA CACAGACTTT

Seladang_11 CGCAAAGAGC CTTACCAGTA TTAAATTTAT CAAAAATTTA AATAACTCAA CACAGACTTT

Seladang_12 CGCAAAGAGC CTTACCAGTA TTAAATTTAT CAAAAATTTA AATAACTCAA CACAGACTTT

Seladang_13 CGCAAAGAGC CTTACCAGTA TTAAATTTAT CAAAAATTTA AATAACTCAA CACAGACTTT

Seladang_14 CGCAAAGAGC CTTACCAGTA TTAAATTTAT CAAAAATTTA AATAACTCAA CACAGACTTT

Seladang_15 CGCAAAGAGC CTTACCAGTA TTAAATTTAT CAAAAATTTA AATAACTCAA CACAGACTTT

Seladang_16 CGCAAAGAGC CTTACCAGTA TTAAATTTAT CAAAAATTTA AATAACTCAA CACAGACTTT

Bison_1 TGCAAAGAGC CTCACCAGTA TTAAATTTAC TAAAAATTCC AATAACTCAA CACAAACTTT

Bubalis_1 TGCAAAGAGC CTTCTCAGTA TCAAATTCAC TAAAACTTGC AACAACTTAA CACTGACTTT

Bubalis_2 TGCAAAGAGC CTTCTCAGTA TTAAATTCAC TAAAACTTGC AACAACTTAA CACTGACTTT

....|....| ....|....| ....|....| ....|....| ....|....| ....|....|

65 75 85 95 105 115

Seladang_A GTACTCTAAC TGAATATCAC AAACGCCACT AAATAACAAC ACACGCCCTA AAACACACCC

Seladang_B GTACTCTAAC TGAATATCAC AAACGCCACT AAATAACAAC ACACGCCCTA AAACACACCC

Seladang_C GTACTCTAAC TGAATATCAC AAACGCCACT AAATAACAAC ACACGCCCTA AAACACACCC

Seladang_D GTACTCTAAC TGAATATCAC AAACGCCACT AAATAACAAC ACACGCCCTA AAACACACCC

Seladang_E GTACTCTAAC TGAATATCAC AAACGCCACT AAATAACAAC ACACGCCCTA AAACACACCC

Seladang_F GTACTCTAAC TGAATATCAC AAACGCCACT AAATAACAAC ACACGCCCTA AAACACACCC

Seladang_G GTACTCTAAC TGAATATCAC AAACGCCACT AAATAACAAC ACACGCCCTA AAACACACCC

Seladang_H GTACTCTAAC TGAATATCAC AAACGCCACT AAATAACAAC ACACGCCCTA AAACACACCC

Seladang_I GTACTCTAAC TGAATATCAC AAACGCCACT AAATAACAAC ACACGCCCTA AAACACACCC

Seladang_J GTACTCTAAC TGAATATCAC AAACGCCACT AAATAACAAC ACACGCCCTA AAACACACCC

Seladang_K GTACTCTAAC TGAATATCAC AAACGCCACT AAATAACAAC ACACGCCCTA AAACACACCC

Seladang_L GTACTCTAAC TGAATATCAC AAACGCCACT AAATAACAAC ACACGCCCTA AAACACACCC

Seladang_M GTACTCTAAC TGAATATCAC AAACGCCACT AAATAACAAC ACACGCCCTA AAACACACCC

Seladang_N GTACTCTAAC TGAATATCAC AAACGCCACT AAATAACAAC ACACGCCCTA AAACACACCC

Seladang_1 GTACTCTAAC TGAATATCAC AAACGCCACT AAATAACAAC ACACGCCCTA AAACACACCC

Seladang_2 GTACTCTAAC TGAATATCAC AAACGCCACT AAATAACAAC ACACGCCCTA AAACACACCC

Seladang_3 GTACTTTAAC TGAATATCAC AAACGCCACT AAATAACAAC ACACGCCCTA AAACACACCC

Seladang_4 GTACTCTAAC TGAATATCAC AAACGCCACT AAATAACAAC ACACGCCCTA AAACACACCC

Seladang_5 GTACTCTAAC TGAATATCAC AAACGCCACT AAATAACAAC ACACGCCCTA AAACACACCC

Seladang_6 GTACTCTAAC TGAATATCAC AAACGCCACT AAATAACAAC ACACGCCCTA AAACACACCC

Seladang_7 GTACTCTAAC TGAATATCAC AAACGCCACT AAATAACAAC ACACGCCCTA AAACACACCC

Seladang_8 GTACTCTAAC TGAATATCAC AAACGCCACT AAATAACAAC ACACGCCCTA AAACACACCC

Seladang_9 GTACTCTAAC TGAATATCAC AAACGCCACT AAAAAACAAC ACACGCCCTA AAACACACCC

Seladang_10 GTACTCTAAC TGAATATCAC AAACGCCACT AAATAACAAC ACACGCCCTA AAACACACCC

Seladang_11 GTACTCTAAC TGAATATCAC AAACGCCACT AAATAACAAC ACACGCCCTA AAACACACCC

Seladang_12 GTACTCTAAC TGAATATCAC AAACGCCACT AAATAACAAC ACACGCCCTA AAACACACCC

Seladang_13 GTACTCTAAC TGAATATCAC AAACGCCACT AAATAACAAC ACACGCCCTA AAACACACCC

Seladang_14 GTACTCTAAC TGAATATCAC AAACGCCACT AAATAACAAC ACACGCCCTA AAACACACCC

Seladang_15 GTACTCTAAC TGAATATCAC AAACGCCACT AAATAACAAC ACACGCCCTA AAACACACCC

Seladang_16 GTACTCTAAC TGAATATCAC AAACGCCACT AAATAACAAC ACACGCCCTA AAACACACCC

Bison_1 GTACTCTAAC CAAATACTGC AAACACCACT AGCTAACGTC ACTCACCCCA AAATGCATTA

Bubalis_1 ACACTCTAGC CTAACATTAG AAATAACTAC AACCATCAAC ACACCTGACA GACCCTACTA

Bubalis_2 ACACTCTAAC CTAACATTAG AAATAACTGC AACCATCAAC ACACCTAACA GGTCTTACCA

....|....| ....|....| ....|....| ....|....| ....|....| ....|....|

125 135 145 155 165 175

Seladang_A TCCAAGCGGA CATAACATTA ATGTAATAAA GACATAATAT GTATATAGTA CATTATATTA

Seladang_B TCCAAGCGGA CATAACATTA ATGTAATAAA GACATAATAT GTATATAGTA CATTATATTA

Seladang_C TCCAAGCGGA CATAACATTA ATGTAATAAA GACATAATAT GTATATAGTA CATTATATTA

Seladang_D TCCAAGCGGA CATAACATTA ATGTAATAAA GACATAATAT GTATATAGTA CATTATATTA

Seladang_E TCCAAGCGGA CATAACATTA ATGTAATAAA GACATAATAT GTATATAGTA CATTATATTA

Seladang_F TCCAAGCGGA CATAACATTA ATGTAATAAA GACATAATAT GTATATAGTA CATTATATTA

Seladang_G TCCAAGCGGA CATAACATTA ATGTAATAAA GACATAATAT GTATATAGTA CATTATATTA

Seladang_H TCCAAGCGGA CATAACATTA ATGTAATAAA GACATAATAT GTATATAGTA CATTATATTA

Seladang_I TCCAAGCGGA CATAACATTA ATGTAATAAA GACATAATAT GTATATAGTA CATTATATTA

Seladang_J TCCAAGCGGA CATAACATTA ATGTAATAAA GACATAATAT GTATATAGTA CATTATATTA

Seladang_K TCCAAGCGGA CATAACATTA ATGTAATAAA GACATAATAT GTATATAGTA CATTATATTA

Seladang_L TCCAAGCGGA CATAACATTA ATGTAATAAA GACATAATAT GTATATAGTA CATTATATTA

Seladang_M TCCAAGCGGA CATAACATTA ATGTAATAAA GACATAATAT GTATATAGTA CATTATATTA

Seladang_N TCCAAGCGGA CATAACATTA ATGTAATAAA GACATAATAT GTATATAGTA CATTATATTA

Seladang_1 TCCAAGCGGA CATAACATTA ATGTAATAAA GACATAATAT GTATATAGTA CATTATATTA

Seladang_2 TCCAAGCGGA CATAACATTA ATGTAATAAA GACATAATAT GTATATAGTA CATTATATTA

Seladang_3 TCCAAGCGGA CATAACATTA ATGTAATAAA GACATAATAT GTATATAGTA CATTATATTA

Seladang_4 TCCAAGCGGA CATAACATTA ATGTAATAAA GACATAATAT GTATATAGTA CATTATATTA

Seladang_5 TCCAAGCGGA CATAACATTA ATGTAATAAA GACATAATAT GTATATAGTA CATTATATTA

Seladang_6 TCCAAGCGGA CATAACATTA ATGTAATAAA GACATAATAT GTATATAGTA CATTATATTA

Seladang_7 TCCAAGCGGA CATAACATTA ATGTAATAAA GACATAATAT GTATATAGTA CATTATATTA

Seladang_8 TCCAAGCGGA CATAACATTA ATGTAATAAA GACATAATAT GTATATAGTA CATTATATTA

Seladang_9 TCCAAGCGGA CATAACATTA ATGTAATAAA GACATAATAT GTATATAGTA CATTATATTA

Seladang_10 TCCAAGCGGA CATAACATTA ATGTAATAAA GACATAATAT GTATATAGTA CATTATATTA

Seladang_11 TCCAAGCGGA CATAACATTA ATGTAATAAA GACATAATAT GTATATAGTA CATTATATTA

Seladang_12 TCCAAGCGGA CATAACATTA ATGTAATAAA GACATAATAT GTATATAGTA CATTATATTA

Seladang_13 TCCAAGCGGA CATAACATTA ATGTAATAAA GACATAATAT GTATATAGTA CATTATATTA

Seladang_14 TCCAAGCGGA CATAACATTA ATGTAATAAA GACATAATAT GTATATAGTA CATTATATTA

Seladang_15 TCCAAGCGGA CATAACATTA ATGTAATAAA GACATAATAT GTATATAGTA CATTATATTA

Seladang_16 TCCAAGCGGA CATAACATTA ATGTAATAAA GACATAATAT GTATATAGTA CATTATATTA

Bison_1 CCCAAACGGA CATAACATTA ATGTAATAAA AACATATTAT GTATATAGTA CATTAAATTA

Bubalis_1 CCCGAATGGA CATAACATTA ATGTAATAAG GACATAATAT GTATATAGTA CATTATATTA

Bubalis_2 CCCGAACGTA CATAACATTA ATGTAACAAG GACATAATAT GTATATAGTA CATTACATTA

....|....| ....|....| ....|....| ....|....| ....|....| ....|....|

185 195 205 215 225 235

Seladang_A TATGCCCCAT GCATATAAAC AAGTACTTGA ACTCATATAG TACATAGTAC ATGAACTTAT

Seladang_B TATGCCCCAT GCATATAAAC AAGTACTTGA ACTCATATAG TACATAGTAC ATGAACTTAT

Seladang_C TATGCCCCAT GCATATAAAC AAGTACTTGA ACTCATATAG TACATAGTAC ATGAACTTAT

Seladang_D TATGCCCCAT GCATATAAAC AAGTACTTGA ACTCATATAG TACATAGTAC ATGAACTTAT

Seladang_E TATGCCCCAT GCATATAAAC AAGTACTTGA ACTCATATAG TACATAGTAC ATGAACTTAT

Seladang_F TATGCCCCAT GCATATAAAC AAGTACTTGA ACTCATATAG TACATAGTAC ATGAACTTAT

Seladang_G TATGCCCCAT GCATATAAAC AAGTACTTGA ACTCATATAG TACATAGTAC ATGAACTTAT

Seladang_H TATGCCCCAT GCATATAAAC AAGTACTTGA ACTCATATAG TACATAGTAC ATGAACTTAT

Seladang_I TATGCCCCAT GCATATAAAC AAGTACTTGA ACTCATATAG TACATAGTAC ATGAACTTAT

Seladang_J TATGCCCCAT GCATATAAAC AAGTACTTGA ACTCATATAG TACATAGTAC ATGAACTTAT

Seladang_K TATGCCCCAT GCATATAAAC AAGTACTTGA ACTCATATAG TACATAGTAC ATGAACTTAT

Seladang_L TATGCCCCAT GCATATAAAC AAGTACTTGA ACTCATATAG TACATAGTAC ATGAACTTAT

Seladang_M TATGCCCCAT GCATATAAAC AAGTACTTGA ACTCATATAG TACATAGTAC ATGAACTTAT

Seladang_N TATGCCCCAT GCATATAAAC AAGTACTTGA ACTCATATAG TACATAGTAC ATGAACTTAT

Seladang_1 TATGCCCCAT GCATATAAAC AAGTACTTGA ACTCATATAG TACATAGTAC ATGAACTTAT

Seladang_2 TATGCCCCAT GCATATAAAC AAGTACTTGA ACTCATATAG TACATAGTAC ATGAACTTAT

Seladang_3 TATGCCCCAT GCATATAAAC AAGTACTTGA ACTCATATAG TACATAGTAC ATGAACTTAT

Seladang_4 TATGCCCCAT GCATATAAAC AAGTACTTGA ACTCATATAG TACATAGTAC ATGAACTTAT

Seladang_5 TATGCCCCAT GCATATAAAC AAGTACTTGA ACTCATATAG TACATAGTAC ATGAACTTAT

Seladang_6 TATGCCCCAT GCATATAAAC AAGTACTTGA ACTCATATAG TACATAGTAC ATGAACTTAT

Seladang_7 TATGCCCCAT GCATATAAAC AAGTACTTGA ACTCATATAG TACATAGTAC ATGAACTTAT

Seladang_8 TATGCCCCAT GCATATAAAC AAGTACTTGA ACTCATATAG TACATAGTAC ATGAACTTAT

Seladang_9 TATGCCCCAT GCATATAAAC AAGTACTTGA ACTCATATAG TACATAGTAC ATGAACTTAT

Seladang_10 TATGCCCCAT GCATATAAAC AAGTACTTGA ACTCATATAG TACATAGTAC ATGAACTTAT

Seladang_11 TATGCCCCAT GCATATAAAC AAGTACTTGA ACTCATATAG TACATAGTAC ATGAACTTAT

Seladang_12 TATGCCCCAT GCATATAAAC AAGTACTTGA ACTCATATAG TACATAGTAC ATGAACTTAT

Seladang_13 TATGCCCCAT GCATATAAAC AAGTACTTGA ACTCATATAG TACATAGTAC ATGAACTTAT

Seladang_14 TATGCCCCAT GCATATAAAC AAGTACTTGA ACTCATATAG TACATAGTAC ATGAACTTAT

Seladang_15 TATGCCCCAT GCATATAAAC AAGTACTTGA ACTCATATAG TACATAGTAC ATGAACTTAT

Seladang_16 TATGCCCCAT GCATATAAAC AAGTACTTGA ACTCATATAG TACATAGTAC ATGAACTTAT

Bison_1 TATGCCCCAT GCATATAAGC AAGTACTTAT CCCTATTCAG TACATAGTAC ATAAAGTTAT

Bubalis_1 TATGCCCCAT GCATATAAGC GGGTACACAA ACTGCATTAG TACATAGTAC ATTCAATTAT

Bubalis_2 TATGCCCCAT GCGTATAAGC AAGTACATAA ACTGCATTAG TACATAGTAC ATACAATTAT

....|....| ....|....| ....|....| ....|....| ....|....| ....|....|

245 255 265 275 285 295

Seladang_A TAATCGTACA TAGCACATTA TGTCAAATTC ATCCTTGGCA ACATGCATAT CCCTTCCACT

Seladang_B TAATCGTACA TAGCACATTA TGTCAAATTC ATCCTTGGCA ACATGCATAT CCCTTCCACT

Seladang_C TAATCGTACA TAGCACATTA TGTCAAATTC ATCCTTGGCA ACATGCATAT CCCTTCCACT

Seladang_D TAATCGTACA TAGCACATTA TGTCAAATTC ATCCTTGGCA ACATGCATAT CCCTTCCACT

Seladang_E TAATCGTACA TAGCACATTA TGTCAAATTC ATCCTTGGCA ACATGCATAT CCCTTCCACT

Seladang_F TAATCGTACA TAGCACATTA TGTCAAATTC ATCCTTGGCA ACATGCATAT CCCTTCCACT

Seladang_G TAATCGTACA TAGCACATTA TGTCAAATTC ATCCTTGGCA ACATGCATAT CCCTTCCACT

Seladang_H TAATCGTACA TAGCACATTA TGTCAAATTC ATCCTTGGCA ACATGCATAT CCCTTCCACT

Seladang_I TAATCGTACA TAGCACATTA TGTCAAATTC ATCCTTGGCA ACATGCATAT CCCTTCCACT

Seladang_J TAATCGTACA TAGCACATTA TGTCAAATTC ATCCTTGGCA ACATGCATAT CCCTTCCACT

Seladang_K TAATCGTACA TAGCACATTA TGTCAAATTC ATCCTTGGCA ACATGCATAT CCCTTCCACT

Seladang_L TAATCGTACA TAGCACATTA TGTCAAATTC ATCCTTGGCA ACATGCATAT CCCTTCCACT

Seladang_M TAATCGTACA TAGCACATTA TGTCAAATTC ATCCTTGGCA ACATGCATAT CCCTTCCACT

Seladang_N TAATCGTACA TAGCACATTA TGTCAAATTC ATCCTTGGCA ACATGCATAT CCCTTCCACT

Seladang_1 TAATCGTACA TAGCACATTA TGTCAAATTC ATCCTTGGCA ACATGCATAT CCCTTCCACT

Seladang_2 TAATCGTACA TAGCACATTA TGTCAAATTC ATCCTTGGCA ACATGCATAT CCCTTCCACT

Seladang_3 TAATCGTACA TAGCACATTA TGTCAAATTC ATCCTTGGCA ACATGCATAT CCCTTCCACT

Seladang_4 TAATCGTACA TAGCACATTA TGTCAAATTC ATCCTTGGCA ACATGCATAT CCCTTCCACT

Seladang_5 TAATCGTACA TAGCACATTA TGTCAAATTC ATCCTTGGCA ACATGCATAT CCCTTCCACT

Seladang_6 TAATCGTACA TAGCACATTA TGTCAAATTC ATCCTTGGCA ACATGCATAT CCCTTCCACT

Seladang_7 TAATCGTACA TAGCACATTA TGTCAAATTC ATCCTTGGCA ACATGCATAT CCCTTCCACT

Seladang_8 TAATCGTACA TAGCACATTA TGTCAAATTC ATCCTTGGCA ACATGCATAT CCCTTCCACT

Seladang_9 TAATCGTACA TAGCACATTA TGTCAAATTC ATCCTTGGCA ACATGCATAT CCCTTCCACT

Seladang_10 TAATCGTACA TAGCACATTA TGTCAAATTC ATCCTTGGCA ACATGCATAT CCCTTCCACT

Seladang_11 TAATCGTACA TAGCACATTA TGTCAAATTC ATCCTTGGCA ACATGCATAT CCCTTCCACT

Seladang_12 TAATCGTACA TAGCACATTA TGTCAAATTC ATCCTTGGCA ACATGCATAT CCCTTCCACT

Seladang_13 TAATCGTACA TAGCACATTA TGTCAAATTC ATCCTTGGCA ACATGCATAT CCCTTCCACT

Seladang_14 TAATCGTACA TAGCACATTA TGTCAAATTC ATCCTTGGCA ACATGCATAT CCCTTCCACT

Seladang_15 TAATCGTACA TAGCACATTA TGTCAAATTC ATCCTTGGCA ACATGCATAT CCCTTCCACT

Seladang_16 TAATCGTACA TAGCACATTA TGTCAAATTC ATCCTTGGCA ACATGCATAT CCCTTCCACT

Bison_1 TAATTGTACA TAGCACATTA TGTCAAATCT ACCCTTGGCA ACATGCATAC CCCTTCCATT

Bubalis_1 TGATCGTACA TAGTGCATTA AGTCAAATCC GTCCTCGCCA ACATGCATAT CCCCTCCACT

Bubalis_2 TGATCGTACA TAGCACATTA AGTCAAATCC ATTCTCATCA ACATGCGTAT CCCTTCCATT

....|....| ....|....| ....|....| ....|....| ....|....| ....|....|

305 315 325 335 345 355

Seladang_A AGATCACGAG CTTAATTACC ATGCCGCGTG AAACCAGCAA CCCGCTAGGC AGGGATCCCT

Seladang_B AGATCACGAG CTTAATTACC ATGCCGCGTG AAACCAGCAA CCCGCTAGGC AGGGATCCCT

Seladang_C AGATCACGAG CTTAATTACC ATGCCGCGTG AAACCAGCAA CCCGCTAGGC AGGGATCCCT

Seladang_D AGATCACGAG CTTAATTACC ATGCCGCGTG AAACCAGCAA CCCGCTAGGC AGGGATCCCT

Seladang_E AGATCACGAG CTTAATTACC ATGCCGCGTG AAACCAGCAA CCCGCTAGGC AGGGATCCCT

Seladang_F AGATCACGAG CTTAATTACC ATGCCGCGTG AAACCAGCAA CCCGCTAGGC AGGGATCCCT

Seladang_G AGATCACGAG CTTAATTACC ATGCCGCGTG AAACCAGCAA CCCGCTAGGC AGGGATCCCT

Seladang_H AGATCACGAG CTTAATTACC ATGCCGCGTG AAACCAGCAA CCCGCTAGGC AGGGATCCCT

Seladang_I AGATCACGAG CTTAATTACC ATGCCGCGTG AAACCAGCAA CCCGCTAGGC AGGGATCCCT

Seladang_J AGATCACGAG CTTAATTACC ATGCCGCGTG AAACCAGCAA CCCGCTAGGC AGGGATCCCT

Seladang_K AGATCACGAG CTTAATTACC ATGCCGCGTG AAACCAGCAA CCCGCTAGGC AGGGATCCCT

Seladang_L AGATCACGAG CTTAATTACC ATGCCGCGTG AAACCAGCAA CCCGCTAGGC AGGGATCCCT

Seladang_M AGATCACGAG CTTAATTACC ATGCCGCGTG AAACCAGCAA CCCGCTAGGC AGGGATCCCT

Seladang_N AGATCACGAG CTTAATTACC ATGCCGCGTG AAACCAGCAA CCCGCTAGGC AGGGATCCCT

Seladang_1 AGATCACGAG CTTAATTACC ATGCCGCGTG AAACCAGCAA CCCGCTAGGC AGGGATCCCT

Seladang_2 AGATCACGAG CTTAATTACC ATGCCGCGTG AAACCAGCAA CCCGCTAGGC AGGGATCCCT

Seladang_3 AGATCACGAG CTTAATTACC ATGCCGCGTG AAACCAGCAA CCCGCTAGGC AGGGATCCCT

Seladang_4 AGATCACGAG CTTAATTACC ATGCCGCGTG AAACCAGCAA CCCGCTAGGC AGGGATCCCT

Seladang_5 AGATCACGAG CTTAATTAAC ATGCCGTGTG AAACCAGCAA CCCGCTAGGC AGGGATCCCT

Seladang_6 AGATCACGAG CTTAATTACC ATGCCGCGTG AAACCAGCAA CCCGCTAGGC AGGGATCCCT

Seladang_7 AGATCACGAG CTTAATTACC ATGCCGCGTG AAACCAGCAA CCCGCTAGGC AGGGATCCCT

Seladang_8 AGATCACGAG CTTAATTACC ATGCCGCGTG AAACCAGCAA CCCGCTAGGC AGGGATCCCT

Seladang_9 AGATCACGAG CTTAATTACC ATGCCGCGTG AAACCAGCAA CCCGCTAGGC AGGGATCCCT

Seladang_10 AGATCACGAG CTTAATTACC ATGCCGCGTG AAACCAGCAA CCCGCTAGGC AGGGATCCCT

Seladang_11 AGATCACGAG CTTAATTACC ATGCCGCGTG AAACCAGCAA CCCGCTAGGC AGGGATCCCT

Seladang_12 AGATCACGAG CTTAATTACC ATGCCGCGTG AAACCAGCAA CCCGCTAGGC AGGGATCCCT

Seladang_13 AGATCACGAG CTTAATTACC ATGCCGCGTG AAACCAGCAA CCCGCTAGGC AGGGATCCCT

Seladang_14 AGATCACGAG CTTAATTACC ATGCCGCGTG AAACCAGCAA CCCGCTAGGC AGGGATCCCT

Seladang_15 AGATCACGAG CTTAATTACC ATGCCGCGTG AAACCAGCAA CCCGCTAGGC AGGGATCCCT

Seladang_16 AGATCACGAG CTTAATTACC ATGCCGCGTG AAACCAGCAA CCCGCTAGGC AGGGATCCCT

Bison_1 AGATCACGAG CTTAATTACC ATGCCGCGTG AAACCAGCAA CCCGCTAGGC AGGGATCCCT

Bubalis_1 AGATCACGAG CTTGGTCACC ATGCCGCGTG AAACCAGCAA CCCTTCAGAC AGGGATCCCT

Bubalis_2 AGATCACGAG CTTGATCACC ATGCCGCGTG AAATCAGCAA CCCTTCAGGC AGGGATCCCT

....|....| ....|....| ....|....| ....|....| ....|....| ....|....|

365 375 385 395 405 415

Seladang_A CTTCTCGCTC CGGGCCCATG AACCGTGGGG GTCGCTATTT AATGAACTTT ATCAGACATC

Seladang_B CTTCTCGCTC CGGGCCCATG AACCGTGGGG GTCGCTATTT AATGAACTTT ATCAGACATC

Seladang_C CTTCTCGCTC CGGGCCCATG AACCGTGGGG GTCGCTATTT AATGAACTTT ATCAGACATC

Seladang_D CTTCTCGCTC CGGGCCCATG AACCGTGGGG GTCGCTATTT AATGAACTTT ATCAGACATC

Seladang_E CTTCTCGCTC CGGGCCCATG AACCGTGGGG GTCGCTATTT AATGAACTTT ATCAGACATC

Seladang_F CTTCTCGCTC CGGGCCCATG AACCGTGGGG GTCGCTATTT AATGAACTTT ATCAGACATC

Seladang_G CTTCTCGCTC CGGGCCCATG AACCGTGGGG GTCGCTATTT AATGAACTTT ATCAGACATC

Seladang_H CTTCTCGCTC CGGGCCCATG AACCGTGGGG GTCGCTATTT AATGAACTTT ATCAGACATC

Seladang_I CTTCTCGCTC CGGGCCCATG AACCGTGGGG GTCGCTATTT AATGAACTTT ATCAGACATC

Seladang_J CTTCTCGCTC CGGGCCCATG AACCGTGGGG GTCGCTATTT AATGAACTTT ATCAGACATC

Seladang_K CTTCTCGCTC CGGGCCCATG AACCGTGGGG GTCGCTATTT AATGAACTTT ATCAGACATC

Seladang_L CTTCTCGCTC CGGGCCCATG AACCGTGGGG GTCGCTATTT AATGAACTTT ATCAGACATC

Seladang_M CTTCTCGCTC CGGGCCCATG AACCGTGGGG GTCGCTATTT AATGAACTTT ATCAGACATC

Seladang_N CTTCTCGCTC CGGGCCCATG AACCGTGGGG GTCGCTATTT AATGAACTTT ATCAGACATC

Seladang_1 CTTCTCGCTC CGGGCCCATG AACCGTGGGG GTCGCTATTT AATGAACTTT ATCAGACATC

Seladang_2 CTTCTCGCTC CGGGCCCATG AACCGTGGGG GTCGCTATTT AATGAACTTT ATCAGACATC

Seladang_3 CTTCTCGCTC CGGGCCCATG AACCGTGGGG GTCGCTATTT AATGAACTTT ATCAGACATC

Seladang_4 CTTCTCGCTC CGGGCCCATG AACCGTGGGG GTCGCTATTT AATGAACTTT ATCAGACATC

Seladang_5 CTTCTCGCTC CGGGCCCATG AACCGTGGGG GTCGCTATTT AATGAACTTT ATCAGACATC

Seladang_6 CTTCTCGCTC CGGGCCCATG AACCGTGGGG GTCGCTATTT AATGAACTTT ATCAGACATC

Seladang_7 CTTCTCGCTC CGGGCCCATG AACCGTGGGG GTCGCTATTT AATGAACTTT ATCAGACATC

Seladang_8 CTTCTCGCTC CGGGCCCATG AACCGTGGGG GTCGCTATTT AATGAACTTT ATCAGACATC

Seladang_9 CTTCTCGCTC CGGGCCCATG AACCGTGGGG GTCGCTATTT AATGAACTTT ATCAGACATC

Seladang_10 CTTCTCGCTC CGGGCCCATG AACCGTGGGG GTCGCTATTT AATGAACTTT ATCAGACATC

Seladang_11 CTTCTCGCTC CGGGCCCATG AACCGTGGGG GTCGCTATTT AATGAACTTT ATCAGACATC

Seladang_12 CTTCTCGCTC CGGGCCCATG AACCGTGGGG GTCGCTATTT AATGAACTTT ATCAGACATC

Seladang_13 CTTCTCGCTC CGGGCCCATG AACCGTGGGG GTCGCTATTT AATGAACTTT ATCAGACATC

Seladang_14 CTTCTCGCTC CGGGCCCATG AACCGTGGGG GTCGCTATTT AATGAACTTT ATCAGACATC

Seladang_15 CTTCTCGCTC CGGGCCCATG AACCGTGGGG GTCGCTATTT AATGAACTTT ATCAGACATC

Seladang_16 CTTCTCGCTC CGGGCCCATG AACCGTGGGG GTCGCTATTT AATGAACTTT ATCAGACATC

Bison_1 CTTCTCGCTC CGGGCCCATG AACCGTGGGG GTCGCTATTT AATGAACTTT ATCAGACATC

Bubalis_1 CTTCTCGCTC CGGGCCCATG TCTTGTGGGG GTAGCTATTC AATGAACTTT AACAGGCATC

Bubalis_2 CTTCTCGCTC CGGGCCCATG TCATGTGGGG GTAGCTACTT AATGAACTTT AACAGACATC

....|....| ....|....| ....|....| ....|....| ....|....| ....|....|

425 435 445 455 465 475

Seladang_A TGGTTCTTTC TTCAGGGCCA TCTCATCTAA AATCGTCCAT TCTTTCCTCT TAAATAAGAC

Seladang_B TGGTTCTTTC TTCAGGGCCA TCTCATCTAA AATCGTCCAT TCTTTCCTCT TAAATAAGAC

Seladang_C TGGTTCTTTC TTCAGGGCCA TCTCATCTAA AATCGTCCAT TCTTTCCTCT TAAATAAGAC

Seladang_D TGGTTCTTTC TTCAGGGCCA TCTCATCTAA AATCGTCCAT TCTTTCCTCT TAAATAAGAC

Seladang_E TGGTTCTTTC TTCAGGGCCA TCTCATCTAA AATCGTCCAT TCTTTCCTCT TAAATAAGAC

Seladang_F TGGTTCTTTC TTCAGGGCCA TCTCATCTAA AATCGTCCAT TCTTTCCTCT TAAATAAGAC

Seladang_G TGGTTCTTTC TTCAGGGCCA TCTCATCTAA AATCGTCCAT TCTTTCCTCT TAAATAAGAC

Seladang_H TGGTTCTTTC TTCAGGGCCA TCTCATCTAA AATCGTCCAT TCTTTCCTCT TAAATAAGAC

Seladang_I TGGTTCTTTC TTCAGGGCCA TCTCATCTAA AATCGTCCAT TCTTTCCTCT TAAATAAGAC

Seladang_J TGGTTCTTTC TTCAGGGCCA TCTCATCTAA AATCGTCCAT TCTTTCCTCT TAAATAAGAC

Seladang_K TGGTTCTTTC TTCAGGGCCA TCTCATCTAA AATCGTCCAT TCTTTCCTCT TAAATAAGAC

Seladang_L TGGTTCTTTC TTCAGGGCCA TCTCATCTAA AATCGTCCAT TCTTTCCTCT TAAATAAGAC

Seladang_M TGGTTCTTTC TTCAGGGCCA TCTCATCTAA AATCGTCCAT TCTTTCCTCT TAAATAAGAC

Seladang_N TGGTTCTTTC TTCAGGGCCA TCTCATCTAA AATCGTCCAT TCTTTCCTCT TAAATAAGAC

Seladang_1 TGGTTCTTTC TTCAGGGCCA TCTCATCTAA AATCGTCCAT TCTTTCCTCT TAAATAAGAC

Seladang_2 TGGTTCTTTC TTCAGGGCCA TCTCATCTAA AATCGTCCAT TCTTTCCTCT TAAATAAGAC

Seladang_3 TGGTTCTTTC TTCAGGGCCA TTTCATCTAA AATCGTCCAT TCTTTCCTCT TAAATAAGAC

Seladang_4 TGGTTCTTTC TTCAGGGCCA TCTCATCTAA AATCGTCCAT TCTTTCCTCT TAAATAAGAC

Seladang_5 TGGTTCTTTC TTCAGGGCCA TCTCATCTAA AATAGTCCAT TCTTTCCTCT TAAATAAGAC

Seladang_6 TGGTTCTTTC TTCAGGGCCA TCTCATCTAA AATCGTCCAT TCTTTCCTCT TAAATAAGAC

Seladang_7 TGGTTCTTTC TTCAGGGCCA TCTCATCTAA AATCGTCCAT TCTTTCCTCT TAAATAAGAC

Seladang_8 TGGTTCTTTC TTCAGGGCCA TCTCATCTAA AATCGTCCAT TCTTTCCTCT TAAATAAGAC

Seladang_9 TGGTTCTTTC TTCAGGGCCA TCTCATCTAA AATCGTCCAT TCTTTCCTCT TAAATAAGAC

Seladang_10 TGGTTCTTTC TTCAGGGCCA TCTCATCTAA AATCGTCCAT TCTTTCCTCT TAAATAAGAC

Seladang_11 TGGTTCTTTC TTCAGGGCCA TCTCATCTAA AATCGTCCAT TCTTTCCTCT TAAATAAGAC

Seladang_12 TGGTTCTTTC TTCAGGGCCA TCTCATCTAA AATCGTCCAT TCTTTCCTCT TAAATAAGAC

Seladang_13 TGGTTCTTTC TTCAGGGCCA TCTCATCTAA AATCGTCCAT TCTTTCCTCT TAAATAAGAC

Seladang_14 TGGTTCTTTC TTCAGGGCCA TCTCATCTAA AATCGTCCAT TCTTTCCTCT TAAATAAGAC

Seladang_15 TGGTTCTTTC TTCAGGGCCA TCTCATCTAA AATCGTCCAT TCTTTCCTCT TAAATAAGAC

Seladang_16 TGGTTCTTTC TTCAGGGCCA TCTCATCTAA AATCGTCCAT TCTTTCCTCT TAAATAAGAC

Bison_1 TGGTTCTTTC TTCGGGGCCA TCTCACCTAA AATCGCCCAT TCTTTCCTCT TAAATAAGAC

Bubalis_1 TGGTTCTTTC TTCAGGGCCA TCTCACCTAA AATCGCCCAC TCTTTCCCCT TAAATAAGAC

Bubalis_2 TGGTTCTTTC TTCAGGGCCA TCTCACCTAA AGTCGCCCAT TCTTTCCTCT TAAATAAGAC

....|....| ....|....| ....|....| ....|....| ....|....| ....|....|

485 495 505 515 525 535

Seladang_A ATCTCGATGG ACTAATGACT AATCAGCCAT GCTCACACAT AACTGTGCTG TCATACATTT

Seladang_B ATCTCGATGG ACTAATGACT AATCAGCCAT GCTCACACAT AACTGTGCTG TCATACATTT

Seladang_C ATCTCGATGG ACTAATGACT AATCAGCCAT GCTCACACAT AACTGTGCTG TCATACATTT

Seladang_D ATCTCGATGG ACTAATGACT AATCAGCCAT GCTCACACAT AACTGTGCTG TCATACATTT

Seladang_E ATCTCGATGG ACTAATGACT AATCAGCCAT GCTCACACAT AACTGTGCTG TCATACATTT

Seladang_F ATCTCGATGG ACTAATGACT AATCAGCCAT GCTCACACAT AACTGTGCTG TCATACATTT

Seladang_G ATCTCGATGG ACTAATGACT AATCAGCCAT GCTCACACAT AACTGTGCTG TCATACATTT

Seladang_H ATCTCGATGG ACTAATGACT AATCAGCCAT GCTCACACAT AACTGTGCTG TCATACATTT

Seladang_I ATCTCGATGG ACTAATGACT AATCAGCCAT GCTCACACAT AACTGTGCTG TCATACATTT

Seladang_J ATCTCGATGG ACTAATGACT AATCAGCCAT GCTCACACAT AACTGTGCTG TCATACATTT

Seladang_K ATCTCGATGG ACTAATGACT AATCAGCCAT GCTCACACAT AACTGTGCTG TCATACATTT

Seladang_L ATCTCGATGG ACTAATGACT AATCAGCCAT GCTCACACAT AACTGTGCTG TCATACATTT

Seladang_M ATCTCGATGG ACTAATGACT AATCAGCCAT GCTCACACAT AACTGTGCTG TCATACATTT

Seladang_N ATCTCGATGG ACTAATGACT AATCAGCCAT GCTCACACAT AACTGTGCTG TCATACATTT

Seladang_1 ATCTCGATGG ACTAATGACT AATCAGCCAT GCTCACACAT AACTGTGCTG TCATACATTT

Seladang_2 ATCTCGATGG ACTAATGACT AATCAGCCAT GCTCACACAT AACTGTGCTG TCATACATTT

Seladang_3 ATCTCGATGG ACTAATGACT AATCAGCCAT GCTCACACAT AACTGTGCTG TCATACATTT

Seladang_4 ATCTCGATGG ACTAATGACT AATCAGCCAT GCTCACACAT AACTGTGCTG TCATACATTT

Seladang_5 ATCTCGATGG ACTAATGACT AATCAGCCAT GCTCACACAT AACTGTGCTG TCATACATTT

Seladang_6 ATCTCGATGG ACTAATGACT AATCAGCCAT GCTCACACAT AACTGTGCTG TCATACATTT

Seladang_7 ATCTCGATGG ACTAATGACT AATCAGCCAT GCTCACACAT AACTGTGCTG TCATACATTT

Seladang_8 ATCTCGATGG ACTAATGACT AATCAGCCAT GCTCACACAT AACTGTGCTG TCATACATTT

Seladang_9 ATCTCGATGG ACTAATGACT AATCAGCCAT GCTCACACAT AACTGTGCTG TCATACATTT

Seladang_10 ATCTCGATGG ACTAATGACT AATCAGCCAT GCTCACACAT AACTGTGCTG TCATACATTT

Seladang_11 ATCTCGATGG ACTAATGACT AATCAGCCAT GCTCACACAT AACTGTGCTG TCATACATTT

Seladang_12 ATCTCGATGG ACTAATGACT AATCAGCCAT GCTCACACAT AACTGTGCTG TCATACATTT

Seladang_13 ATCTCGATGG ACTAATGACT AATCAGCCAT GCTCACACAT AACTGTGCTG TCATACATTT

Seladang_14 ATCTCGATGG ACTAATGACT AATCAGCCAT GCTCACACAT AACTGTGCTG TCATACATTT

Seladang_15 ATCTCGATGG ACTAATGACT AATCAGCCAT GCTCACACAT AACTGTGCTG TCATACATTT

Seladang_16 ATCTCGATGG ACTAATGACT AATCAGCCAT GCTCACACAT AACTGTGCTG TCATACATTT

Bison_1 ATCTCGATGG ACTAATGGCT AATCAGCCAT GCTCACACAT AACTGTGCTG TCATACATTT

Bubalis_1 ATCTCGATGG ACTAATGTCT AATCAGCCAT GCTCACACAT AACTGTGCTG TCATACATTT

Bubalis_2 ATCTCGATGG ACTAATGTCT AATCAGCCAT GCTCACACAT AACTGTGCTG TCATACATTT

....|....| ....|....| ....|....| ....|....| ....|....| ....|....|

545 555 565 575 585 595

Seladang_A GGTATTTTTT TATTTTGGGG GATGCTTGGA CTCAGCTATG GCCGTCAAAG GCCCGACCCG

Seladang_B GGTATTTTTT TATTTTGGGG GATGCTTGGA CTCAGCTATG GCCGTCAAAG GCCCGACCCG

Seladang_C GGTATTTTTT TATTTTGGGG GATGCTTGGA CTCAGCTATG GCCGTCAAAG GCCCGACCCG

Seladang_D GGTATTTTTT TATTTTGGGG GATGCTTGGA CTCAGCTATG GCCGTCAAAG GCCCGACCCG

Seladang_E GGTATTTTTT TATTTTGGGG GATGCTTGGA CTCAGCTATG GCCGTCAAAG GCCCGACCCG

Seladang_F GGTATTTTTT TATTTTGGGG GATGCTTGGA CTCAGCTATG GCCGTCAAAG GCCCGACCCG

Seladang_G GGTATTTTTT TATTTTGGGG GATGCTTGGA CTCAGCTATG GCCGTCAAAG GCCCGACCCG

Seladang_H GGTATTTTTT TATTTTGGGG GATGCTTGGA CTCAGCTATG GCCGTCAAAG GCCCGACCCG

Seladang_I GGTATTTTTT TATTTTGGGG GATGCTTGGA CTCAGCTATG GCCGTCAAAG GCCCGACCCG

Seladang_J GGTATTTTTT TATTTTGGGG GATGCTTGGA CTCAGCTATG GCCGTCAAAG GCCCGACCCG

Seladang_K GGTATTTTTT TATTTTGGGG GATGCTTGGA CTCAGCTATG GCCGTCAAAG GCCCGACCCG

Seladang_L GGTATTTTTT TATTTTGGGG GATGCTTGGA CTCAGCTATG GCCGTCAAAG GCCCGACCCG

Seladang_M GGTATTTTTT TATTTTGGGG GATGCTTGGA CTCAGCTATG GCCGTCAAAG GCCCGACCCG

Seladang_N GGTATTTTTT TATTTTGGGG GATGCTTGGA CTCAGCTATG GCCGTCAAAG GCCCGACCCG

Seladang_1 GGTATTTTTT TATTTTGGGG GATGCTTGGA CTCAGCTATG GCCGTCAAAG GCCCGACCCG

Seladang_2 GGTATTTTTT TATTTTGGGG GATGCTTGGA CTCAGCTATG GCCGTCAAAG GCCCGACCCG

Seladang_3 GGTATTTTTT TATTTTGGGG GATGCTTGGA CTCAGCTATG GCCGTCAAAG GCCCGACCCG

Seladang_4 GGTATTTTTT TATTTTGGGG GATGCTTGGA CTCAGCTATG GCCGTCAAAG GCCCGACCCG

Seladang_5 GGTATTTTTT TATTTTGGGG GATGCTTGGA TTCAGCTATG GCCGTCAAAG GCCCGACCCG

Seladang_6 GGTATTTTTT TATTTTGGGG GATGCTTGGA CTCAGCTATG GCCGTCAAAG GCCCGACCCG

Seladang_7 GGTATTTTTT TATTTTGGGG GATGCTTGGA CTCAGCTATG GCCGTCAAAG GCCCGACCCG

Seladang_8 GGTATTTTTT TATTTTGGGG GATGCTTGGA CTCAGCTATG GCCGTCAAAG GCCCGACCCG

Seladang_9 GGTATTTTTT TATTTTGGGG GATGCTTGGA CTCAGCTATG GCCGTCAAAG GCCCGACCCG

Seladang_10 GGTATTTTTT TATTTTGGGG GATGCTTGGA CTCAGCTATG GCCGTCAAAG GCCCGACCCG

Seladang_11 GGTATTTTTT TATTTTGGGG GATGCTTGGA CTCAGCTATG GCCGTCAAAG GCCCGACCCG

Seladang_12 GGTATTTTTT TATTTTGGGG GATGCTTGGA CTCAGCTATG GCCGTCAAAG GCCCGACCCG

Seladang_13 GGTATTTTTT TATTTTGGGG GATGCTTGGA CTCAGCTATG GCCGTCAAAG GCCCGACCCG

Seladang_14 GGTATTTTTT TATTTTGGGG GATGCTTGGA CTCAGCTATG GCCGTCAAAG GCCCGACCCG

Seladang_15 GGTATTTTTT TATTTTGGGG GATGCTTGGA CTCAGCTATG GCCGTCAAAG GCCCGACCCG

Seladang_16 GGTATTTTTT TATTTTGGGG GATGCTTGGA CTCAGCTATG GCCGTCAAAG GCCCGACCCG

Bison_1 GGTATTTTTT TATTTTGGGG GATGCTTGGA CTCAGCTATG GCCGTCAAAG GCCTGACCCG

Bubalis_1 GGTATTTTTT TATTTTGGGG GATGCTTGGA CTCAGCTATG GCCGTCAAAG GCCCGACCCG

Bubalis_2 GGTATTTTTT TATTTTGGGG GATGCTTGGA CTCAGCTATG GCCGTCAAAG GCCCGACCCG

....|....| ....|....| ....|....| ....|....| ....|....| ..

605 615 625 635 645

Seladang_A GAGCATATAT TGTAGCTGGA CTTAACTGCA TCTTGAGCAC CAGCATAATG AT

Seladang_B GAGCATATAT TGTAGCTGGA CTTAACTGCA TCTTGAGCAC CAGCATAATG AT

Seladang_C GAGCATATAT TGTAGCTGGA CTTAACTGCA TCTTGAGCAC CAGCATAATG AT

Seladang_D GAGCATATAT TGTAGCTGGA CTTAACTGCA TCTTGAGCAC CAGCATAATG AT

Seladang_E GAGCATATAT TGTAGCTGGA CTTAACTGCA TCTTGAGCAC CAGCATAATG AT

Seladang_F GAGCATATAT TGTAGCTGGA CTTAACTGCA TCTTGAGCAC CAGCATAATG AT

Seladang_G GAGCATATAT TGTAGCTGGA CTTAACTGCA TCTTGAGCAC CAGCATAATG AT

Seladang_H GAGCATATAT TGTAGCTGGA CTTAACTGCA TCTTGAGCAC CAGCATAATG AT

Seladang_I GAGCATATAT TGTAGCTGGA CTTAACTGCA TCTTGAGCAC CAGCATAATG AT

Seladang_J GAGCATATAT TGTAGCTGGA CTTAACTGCA TCTTGAGCAC CAGCATAATG AT

Seladang_K GAGCATATAT TGTAGCTGGA CTTAACTGCA TCTTGAGCAC CAGCATAATG AT

Seladang_L GAGCATATAT TGTAGCTGGA CTTAACTGCA TCTTGAGCAC CAGCATAATG AT

Seladang_M GAGCATATAT TGTAGCTGGA CTTAACTGCA TCTTGAGCAC CAGCATAATG AT

Seladang_N GAGCATATAT TGTAGCTGGA CTTAACTGCA TCTTGAGCAC CAGCATAATG AT

Seladang_1 GAGCATATAT TGTAGCTGGA CTTAACTGCA TCTTGAGCAC CAGCATAATG AT

Seladang_2 GAGCATATAT TGTAGCTGGA CTTAACTGCA TCTTGAGCAC CAGCATAATG AT

Seladang_3 GAGCATATAT TGTAGCTGGA CTTAACTGCA TCTTGAGCAC CAGCATAATG AT

Seladang_4 GAGCATATAT TGTAGCTGGA CTTAACTGCA TCTTGAGCAC CAGCATAATG AT

Seladang_5 GAGCATATAT TGTAGCTGGA CTTAACTGCA TCTTGAGCAC CAGCATAATG AT

Seladang_6 GAGCATATAT TGTAGCTGGA CTTAACTGCA TCTTGAGCAC CAGCATAATG AT

Seladang_7 GAGCATATAT TGTAGCTGGA CTTAACTGCA TCTTGAGCAC CAGCATAATG AT

Seladang_8 GAGCATATAT TGTAGCTGGA CTTAACTGCA TCTTGAGCAC CAGCATAATG AT

Seladang_9 GAGCATATAT TGTAGCTGGA CTTAACTGCA TCTTGAGCAC CAGCATAATG AT

Seladang_10 GAGCATATAT TGTAGCTGGA CTTAACTGCA TCTTGAGCAC CAGCATAATG AT

Seladang_11 GAGCATATAT TGTAGCTGGA CTTAACTGCA TCTTGAGCAC CAGCATAATG AT

Seladang_12 GAGCATATAT TGTAGCTGGA CTTAACTGCA TCTTGAGCAC CAGCATAATG AT

Seladang_13 GAGCATATAT TGTAGCTGGA CTTAACTGCA TCTTGAGCAC CAGCATAATG AT

Seladang_14 GAGCATATAT TGTAGCTGGA CTTAACTGCA TCTTGAGCAC CAGCATAATG AT

Seladang_15 GAGCATATAT TGTAGCTGGA CTTAACTGCA TCTTGAGCAC CAGCATAATG AT

Seladang_16 GAGCATATAT TGTAGCTGGA CTTAACTGCA TCTTGAGCAC CAGCATAATG AT

Bison_1 GAGCATCTAT TGTAGCTGGA CTTAACTGCA CCTTGAGCAC CAGCATAATG GT

Bubalis_1 GAGCATGAAT TGTAGCTGGA CTTAACTGCA TCTTGAGCAC CAGCATAATG GT

Bubalis_2 GAGCATAAAT TGTAGCTGGA CTTAACTGCA TCTTGAGCAC CAGCATAATG GT
