# Supplementary material for: Sequence variation data of the mitochondrial DNA D-loop region of the captive Malayan Gaur (Bos gaurus hubbacki)
Source: Data Brief. 2018 Nov 27;24:103532. doi: 10.1016/j.dib.2018.11.117 (PMC6531834; doi:10.1016/j.dib.2018.11.117)
Supplement: Supplementary file 1 — Supplementary material. [file mmc1.doc]

CONFLICT OF INTEREST STATEMENT

Prof Dr Badrul Munir Md Zain,	20 NOV 2018

School of Environmental and Natural Resources Sciences,

Faculty of Science and Technology,

Universiti Kebangsaan Malaysia,

43600, Bangi, Selangor,

Malaysia.

Email: [abgbadd@ukm.edu.my](mailto:abgbadd@ukm,.edu.my)	abgbadd1966@yahoo.com

Subject: CONFLICT OF INTEREST STATEMENT

Dear Editor

I am enclosing herewith a manuscript entitled **Sequence Variation Data of the Mitochondrial DNA D-loop region of the Captive Malayan Gaur (*Bos gaurus hubbacki*)** for publication in **Data in Brief**

**I declare no conflict interest exist.**

With the submission of this manuscript I would like to undertake that the above mentioned manuscript has not been published elsewhere, accepted for publication elsewhere or under editorial review for publication elsewhere; and that my Institute’s (**Universiti Kebangsaan Malaysia**) representative is fully aware of this submission.

Yours sincerely,

Prof Dr Badrul Munir Md Zain.

Faculty of Science and Technology,

Universiti Kebangsaan Malaysia,
